# Supplementation of L-carnitine during in vitro maturation of mouse oocytes affects expression of genes involved in oocyte and embryo competence: An experimental study

**Published:** 2017-12

**Authors:** Zohreh Zare, Beheshteh Abouhamzeh, Reza Masteri Farahani, Mohammad Salehi, Moslem Mohammadi

**Affiliations:** 1 *Department of Anatomical Sciences, School of Medicine, Molecular and Cell Biology Research Center, Mazandaran University of Medical Sciences, Sari, Iran.*; 2 *Department of Anatomical Sciences, School of Medicine, AJA University of Medical Sciences, Tehran, Iran.*; 3 *Department of Biology and Anatomical Sciences, School of Medicine, Shahid Beheshti University of Medical Sciences, Tehran, Iran.*; 4 *Department of Biotechnology, School of Advanced Technologies in Medicine, Shahid Beheshti University of Medical Sciences, Tehran, Iran.*; 5 *Department of Physiology and Pharmacology, Molecular and Cell Biology Research Center, School of Medicine, Mazandaran University of Medical Sciences, Sari, Iran.*

**Keywords:** L-carnitine, In vitro fertilization, Oocyte, Gene expression

## Abstract

**Background:**

Oocyte developmental competence is one of the key factors for determining the success rate of assisted reproductive technique.

**Objective:**

The aim of the current study was to investigate the effect of L-carnitine (LC) supplementation during in vitro maturation (IVM), on preimplantation embryo development and expression of genes involved in embryo competence derived from oocytes selected with brilliant cresyl blue (BCB) test.

**Materials and Methods:**

Cumulus-oocyte complexes (COCs) were obtained from NMRI mice ovaries. COCs were stained with BCB and then BCB+ (colored cytoplasm) oocytes cultured in IVM medium supplemented with 0.3 or 0.6 mg/ml LC. COCs untreated with LC were used as control. Fertilization rate and blastocyst development rate were determined after in vitro fertilization. In addition, quantitative reverse transcriptase polymerase chain reaction was used to measure relative genes expression related with development (Ccnb1, Mos, Ces5, and Dppa2) and apoptosis (Bax and Bcl-xL) in oocytes and embryos.

**Results:**

Oocytes treated with both LC concentrations showed higher blastocyst development rate compared with untreated oocytes (p<0.01). Moreover, fertilization rate was increased in oocytes treated with 0.6 mg/ml LC (p<0.01). Treatment of oocytes with both LC concentrations increased (p<0.01) the level of Ccnb1 mRNA in MII oocytes. The two-cell stage embryos and blastocysts derived from LC-treated oocytes (0.6 mg/ml) showed increased the expression levels of Dppa2 and Bcl-xl mRNA, respectively (p<0.01).

**Conclusion:**

The results of the present study show that adding of LC to the IVM medium of BCB+ oocytes can ameliorate reproductive success following in vitro fertilization.

## Introduction

In vitro maturation (IVM) of oocytes has been proposed as an option for infertility treatment (1). Despite the increasing application of IVM in recent years, reduced meiotic maturation and low embryos production can lead to developmental inadequacy of oocytes when compared with in vivo-matured oocytes (2).

Selection of the best quality oocytes is a determining factor in successful IVM and subsequent IVF program (3). A reliable test for selecting fully grown oocytes is brilliant cresyl blue (BCB) staining. BCB, a vital dye, determines the intracellular activity of glucose-6-phosphate dehydrogenase. During the oocytes growth phase, this enzyme is synthesized and then in fully grown oocytes, its activity is declined (4, 5). 

Glucose-6-phosphate dehydrogenase converts BCB from blue to a colorless state, making the cytoplasm of fully grown oocytes into blue color (BCB+) (6). In this regard, previous studies have shown that BCB staining can help selecting competent oocytes, in terms of nuclear maturation and development, in some species, such as pig, sheep, and bovine (7-9). 

Oxidative stress may have detrimental effects on oocyte, fertilization process, and subsequent embryo development (10, 11). L-carnitine (LC) is an endogenous substance which promotes transportation of fatty acids from the cytosol into the mitochondria for beta-oxidation and finally ATP production required for embryo development (12). Besides its significant role in the lipid metabolism, LC has protective effects on the cell through its antioxidant (free radical scavenger), anti-cytokine, and anti-apoptotic actions (13-15). It has been demonstrated that LC supplementation in either maturation or culture media can enhance maturation and developmental potential of camel oocytes (16). LC presumably has beneficial effects in improving yield of assisted reproductive technologies (17, 18). 

The oocyte is very sensitive to changes in its micro-environment (19). Expression of many transcripts in oocytes may be up- or down-regulated with changes in culture media and conditions that affect maturation, fertilization, and embryo viability (20). Establishment of an in vitro maturation system and selection of good quality oocytes can have important implications for improving IVF and related techniques. 

Therefore, the aim of this research was to evaluate the effect of LC supplementation during IVM of mouse immature oocytes, selected with BCB staining, on fertilization rate, preimplantation development, and the expression of genes involved in cell cycle regulation in mature oocytes (Ccnb1 and Mos), the maternal-effect genes in two-cell stage embryos (Ces5 and Dppa2), and the anti- and pro-apoptotic genes in blastocysts derived from BCB+ oocytes (Bcl-xL and Bax, respectively).

## Materials and methods

**Chemicals**

All reagents used in this study were purchased from Sigma-Aldrich Co. (St. Louis, MO, USA), unless specified.

**Animals**

In this experimental study, 80 NMRI mice (female, 6-8 wk and male, 8-12 wk) were obtained from Pasteur Institute, Tehran, Iran). Animals were housed in a temperature- controlled room with a 12 hr light/dark cycle and free access to food and water. 

**Collection of oocytes and BCB staining**

Forty eight hr after superovulation with an intraperitoneal injection of 10 IU pregnant mareʼs serum gonadotropin, mice were sacrificed by cervical dislocation. Cumulus-oocyte complexes (COCs) were mechanically isolated from ovaries by puncturing antral follicles with 26-gauge needles. Almost 25-30 COCs were obtained from the ovaries of each mouse (at least 240 COCs/group). COCs were incubated in potassium simplex optimized medium (KSOM) supplemented with 0.04 mg/ml bovine serum albumin (BSA) containing 26 μM BCB for 90 min at 37^o^C in the presence of 6% CO_2_ under maximum humidity (4). BCB+ oocytes, colored cytoplasm, were selected.

**In vitro oocyte maturation and embryo production**

Procedure for IVM of BCB+ oocytes was that described previously, with some modifications (21). Briefly, oocytes were cultured in IVM medium (TCM 199, with Earle’s salt and L- glutamine, Gibco, Life Tech. Co., UK. enriched with, 24.2 mg/l sodium pyruvate, 1 µg/ml 17-β estradiol, 10 µg/ml LH, 10% FBS, and 10 µg/ml FSH) supplemented with 0.3 or 0.6 mg/ml LC, dissolved in TCM- 199. In control group, oocytes were not treated with LC. The concentrations of LC were selected based on a previous research (22). 

Oocytes were cultured for 24 hr at 37^o^C in the presence of 6% CO_2_ under maximum humidity. Thereafter, the nuclear maturation of oocytes was assessed using 10 μg/ml Hoechst 33342 under an inverted fluorescent microscope (Olympus, Tokyo, Japan). Percentage of metaphase I (MI, containing a metaphase plate, but without a polar body) and metaphase II (MII, containing a metaphasic plate and polar body) oocytes were measured. For sperm preparation, cauda epididymis of male mice was placed in 1 ml Ham's F10 medium supplemented with 5% BSA for 45 min. After maturation, MII oocytes (n=10) along with 25 µl of sperm suspension were added to the 100 µl droplet of fertilization medium (KSOM 15 mg/ml with BSA) and co-incubated for 6 hr at 37^o^C in a humidified atmosphere of 5% CO_2_. In each fertilization droplet, the final concentration of spermatozoa was about 3×106 sperm/ml (23). Fertilization rate was recorded as the percentage of 2-pronucleus embryos from the inseminated oocytes. The zygotes were cultured in 5% CO_2_ incubator at 37^o^C for 5 days. After this time, blastocyst development rate (BDR) was recorded.

**Evaluation of blastocyst quality by differential staining**

On the 5^th^ day of embryo culture, differential staining method was used to count of trophectoderm (TE) and inner cell mass (ICM) cells (24). After washing of blastocysts and hatching blastocysts in flushing holding medium (FHM), zona pellucida was removed via incubation for 45 sec in Tyrode’s solution. The embryos exposed for 30 min to a 1:4 dilution of rabbit anti-mouse serum in FHM. Thereafter, they were incubated in 30% guinea pig complement in FHM supplemented with 10 μg/ml propidium iodide (PI) and 10μg/ml Hoechst 33342 for 30 min. Labeling of propidium iodide and Hoechst nuclei was observed using an epifluorescence microscope with an ultraviolet filter at ×400 magnification. Each embryo was assayed for the number of TE cells (pink or red nuclei) and the number of ICM (blue nuclei).

**Total RNA extraction, cDNA synthesis, and quantitative reverse transcriptase polymerase chain reaction (qRT-PCR)**

Reverse transcription of total RNA was performed using the cDNA synthesis kit (Thermo Fisher Scientific) primed with random hexamer primer according to the manufacturer’s protocol. Three pools of biological replicates (each replicate containing ten denuded mature oocytes, 5 two-cell stage embryos, or 2 blastocysts) were used. PCR was performed in triplicate for each sample. Primer sequences are listed in table I. Amplification of cDNA was carried out in a final volume of 13 µl of PCR mixture, consisted of 1 µl of the cDNA, 1 µl of forward and reverse primers, 6.5 μl of DNA Master SYBR Green I (Roche Applied Sciences), and 4.5 μl H_2_O using a Rotor Gene Q instrument (QIAGEN). The program was initial denaturation at 94^o^C (3 min), 40 cycles of 94^o^C (30 sec), 60^o^C (45 sec), and 72^o^C (45 sec). Hypoxanthine-guanine phospho-ribosyl-transferase (Hprt) was used as an internal reference gene (25). Results were then normalized with Hprt, calculated using 2^-ΔΔCt^, and expressed as relative fold change compared with controls (26).

**Ethical consideration**

All procedures were carried out in accordance with standards for care and use of laboratory animals approved by Ethical Committee of the Shahid Beheshti University of Medical Sciences, Tehran, Iran (SBMU.REC.1392.341).

**Statistical analysis**

Data were analyzed using the SPSS software (Statistical Package for the Social Sciences, version 19.0, SPSS Inc, Chicago, Illinois, USA). Results were presented as mean±SEM. The relative expression of genes, number of ICM and TE were analyzed using one-way analysis of variance (ANOVA), followed by Tukey’s post-hoc test for multiple comparisons. Comparison of maturation rate, fertilization rate, and BDR between groups was performed by Chi-square test. p<0.05 was considered to indicate statistical significance.

**Table I:** Primers used for qRT-PCR experiments

**Gene name**	**Primer sequences (5'- 3')**	**Product size (bp)**	**Accession number**
Hprt	Forward: TCCCAGCGTCGTGATTAG Reverse: CGAGCAAGTCTTTCAGTCC	138	NM_013556.2
Ccnb1	Forward: AAGTCGGAGAGGTTGACG Reverse: TGTCCATTCACCGTTGTC	155	NM_172301.3
Mos	Forward: TCTACACCAAGTCATCTACGG Reverse: AAAATGTTCGCTGGCTTC	168	NM_020021.2
Dppa2	Forward: GACTTTCAACGAGAACCAATC Reverse: GCCTCACCAGAGAACGG	184	NM_007659.3
Ces5	Forward: CATATTGCCCGTGAGAGAC Reverse: CAAACAAGTCCAGTAAAGTG	179	NM_001003951.2
Bax	Forward: CAGCGGCAGTGATGGAC Reverse: TCCTGGATGAAACCCTGTAG	109	NM_007527.3
Bcl-xL	Forward: CAGTCAGCCAGAACCTTATC Reverse: ACACCTGCTCACTTACTGG	83	NM_009743.4

Mos: moloney sarcoma oncogene

Dppa2: developmental pluripotency associated 2

Ces5: carboxylesterase 5

Bax: BCL2-associated X protein

Bcl-xL: BCL2-like 1

## Results

**Effect of LC on oocyte maturation**

To determine if LC treatment alters the maturation rate of BCB+ oocytes, COCs were matured in vitro for 24 hr with/out 0.3 or 0.6 mg/ml LC, as described previously. As shown in table II, LC caused an increase (p<0.01) in maturation rate (75.8%±2 and 76.8%±2 in 0.3 and 0.6 mg/ml LC groups, respectively) as compared with untreated group (56.8%±2.8).

**Effect of LC on embryo development potential**

The effect of LC supplementation during IVM of BCB+ oocytes on fertilization rate and BDR are presented in table III. Adding 0.3 mg/ml of LC to IVM medium did not significantly affect the fertilization rate. The fertilization rate was significantly higher (p<0.01) in 0.6 mg/ml LC group compared with control group. Supplementation of IVM medium with 0.3 and 0.6 mg/ml LC significantly increased (p<0.01) the BDR. There was no significant effect of LC supplementation to IVM medium on the number of ICM and TE in blastocysts.

**Effect of LC on the expression of maturation-related genes in oocytes**

**Figure 1:**
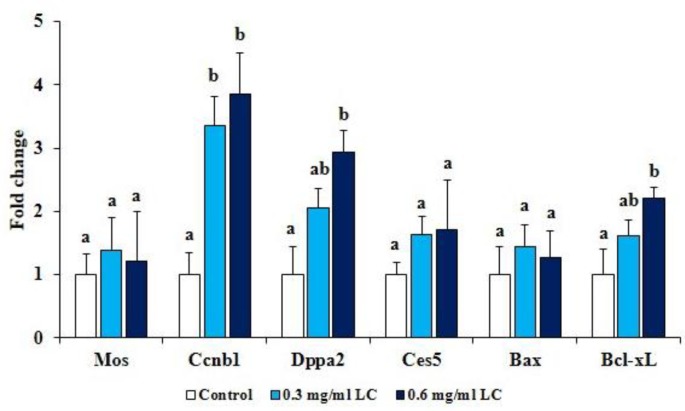
Effect of L-carnitine treatment during IVM on mRNA expression of Mos and Ccnb1 in MII oocytes; Dppa2 and Ces5 in two-cell stage embryos; and Bax and Bcl-xL in blastocysts. Different superscript letters for the same gene demonstrate a significant difference (p<0.01). Values represent mean±SEM of four experiments run in triplicate

The results of qRT-PCR showed that in mature oocytes, the expression of Mos in both LC treated groups were higher than untreated group, although the differences were not statistically significant (Figure 1). However, treatment with both LC concentrations enhanced the expression of Ccnb1 compared with untreated group (p<0.01).

**Effect of LC on the expression of maternal-effect genes in embryos**

Maternal gene expression of Dppa2 and Ces5 in two-cell stage embryos was determined using qRT-PCR (Figure 1). Adding 0.3 mg/ml LC in IVM medium showed no significant effect on the expression of Dppa2. However, 0.6 mg/ml LC enhanced the mRNA expression of Dppa2 in two-cell stage embryos compared with untreated group (p<0.01). Adding 0.3 or 0.6 mg/ml LC to IVM medium had no effect on the mRNA expression of Ces5 in two-cell stage embryos.

**Effect of LC on the expression of apoptosis-related genes in embryos**

The expression of Bcl-xL and Bax were assessed in blastocysts. The results showed that LC treatment (0.3 and 0.6 mg/ml) during IVM of oocytes did not affect the mRNA level of Bax when compared with untreated group. Also, treatment of oocytes with LC (0.6 mg/ml) during IVM increased (p<0.01) the expression of Bcl-xL within blastocysts (Figure 1).

**Table II:** Effect of LC treatment during IVM on nuclear maturation of BCB+ oocytes

**Groups**	**No. of COCs**	**MI n (%)**	**MII n (%)**
Control	243	59 (24.3 ± 1.9)	138 (56.8 ± 2.8)^ a^
0.3 mg/ml LC	285	56 (19.6 ± 2.1)	216 (75.8 ± 2) ^b^
0.6 mg/ml LC	289	60 (20.1 ± 1.7)	229 (76.8 ± 2) ^b^

Values represent mean±SEM of four experiments. LC: L-carnitine

Different superscript letters (a and b) within a column demonstrate a significant difference (p<0.01).

COC: cumulus-oocyte complex

MI: metaphase I oocyte

MII: metaphase II oocyte

**Table III:** Effect of LC treatment during IVM of BCB+ oocytes on embryonic development after IVF

**Groups**	**Inseminated oocytes (n)**	**No. of fertilized oocytes ** **(% fertilization rate)**	**No. of blastocysts (%BDR)**	**No. of ICM**	**No. of TE**
Control	215	140 (66.5 ± 3.3)^ a^	30 (21.8 ± 1.8)^a^	18.4 ± 1.1	36.6 ± 1.5
0.3 mg/ml LC	240	175 (74.6 ± 3.9) ^a^	52 (29.2 ± 1)^b^	20.4 ± 1.2	37.8 ± 1.3
0.6 mg/ml LC	258	214 (83.7 ± 2.1) ^b^	63 (29.8 ± 1.2)^b^	20.9 ± 0.8	37.4 ± 1.3

Values represent mean±SEM of four experiments. Different superscript letters (a and b) within a column demonstrate a significant difference (p<0.01).

BDR: Blastocyst development rate

ICM: Inner cell mass

TE: Trophectoderm

IVM: In vitro maturation

LC: L-carnitine

## Discussion

In the present study, we examined the effects of LC supplementation in the IVM medium of mouse BCB+ oocytes on preimplantation embryonic development. We have shown that LC effectively increased maturation rate of oocytes, fertilization rate, and BDR. In addition, LC improved expression levels of Ccnb1, Dppa2, and Bcl-xL mRNA in MII oocytes, two-cell stage embryos, and blastocysts, respectively. 

Oocyte quality is an important factor in success of the assisted reproductive techniques. Moreover, it has been shown that existence of non-competent oocytes during IVM could negatively affect on developmental competence of oocytes and embryos (27). Among the vital analytic methods used for selecting competent oocytes, BCB staining is a useful technique and shows consistent results (28). It has been demonstrated that selection of competent immature oocytes with BCB test before IVM is efficient method, thus yielding higher in vitro blastocyst development (29).

On the other hand, the culture conditions of oocytes during IVM are critical factors for the successful development of in vitro-produced embryos (30). Also, development of a culture system that meets the requirements of the oocyte during IVM is crucial in order to produce a sufficient number of oocytes to be used clinically. LC is an antioxidant and has an important role in the transportation of long-chain fatty acids into the mitochondria for beta-oxidation and ATP production. ATP levels of oocytes may be responsible for oocyte maturation and developmental capacity of embryos after IVF (31). It was reported that LC has beneficial effects on embryonic development in the mouse, bovine, and porcine (30, 32, 33). We found that compared to control conditions, supplementation of IVM medium with LC significantly increased nuclear maturation rate. 

Numerous factors are involved in cell cycle regulation. During oocyte maturation, accumulation of the proto-oncogene protein, Mos, activates mitogen-activated protein kinase (MAPK). During oocyte maturation, MAPK and maturation promoting factor (MPF) have critical functions in the adjustment of maternal gene expression. MPF is a complex protein, composed of two subunits: cyclin B or Ccnb1, and cdc2 (34). It has been shown that BCB+ oocytes possess the highest phosphorylation rate of MAPKs in MII stage, which may have positive effects on their developmental competence (6). Also, BCB+ oocytes showed a higher number of active mitochondria and MPF activity (8). We previously observed an increase in maturation rate, intracellular level of GSH, and mRNA expression of MAPK1 and CDK1 in mouse BCB+ oocytes after treatment with LC during IVM (23). The results of the present study demonstrated that adding of LC in the IVM medium of mouse BCB+ oocytes increased the Ccnb1 mRNA levels. It could be possible that the higher level of Ccnb1 mRNA may be due to improved oocyte maturation in these oocytes.

Oocyte competence, fertilization, and embryo quality are associated with the meiotic, cytoplasmic, and molecular maturation processes of the oocyte (35). Based on our results, supplementation of BCB+ oocytes with LC during IVM improved fertilization rate and BDR, but it had no significant effect on the number of ICM and TE in the blastocyst. Consistent with our findings, treatment of bovine oocytes with LC (0.6 mg/ml) during IVM improved developmental potential of oocytes and embryos; however, the number and ratio of inner cell mass and trophectoderm cells in blastocysts were unaltered when compared to the control (36). In contrast, treatment of bovine oocytes with different antioxidants such as the vitamin C, resveratrol, or carnitine during IVM induced an increase in total cell number in blastocysts (37). Supplementation of IVM medium with antioxidant reduces oxidative stress-induced embryotoxicity and alters the level of antioxidant enzymes (24). 

Zygote gene activation depends on maternal transcripts and proteins that stored in oocyte during oogenesis and encoded by maternal-effect genes. These maternal products may activate the embryonic genomes and play fundamental roles at the maternal-zygotic stage transition in early embryonic development (38). In the present work, we measured the expression of the two maternal-effect genes (Ces5 and Dppa2) in two-cell stage embryos. Dppa2 (an Oct-4 related gene) is important for embryonic development (39). Our results showed that exposure of BCB+ oocytes during oocyte maturation to LC (0.6 mg/ml) significantly enhanced the expression of Dppa2 in these embryos. Although it is not clear how LC supplementation during IVM of immature oocytes can increase Dppa2 mRNA level, it can be concluded that improved maturation rate of oocytes and increased expression of Dppa2 may have a positive effect on developmental competence of these embryos.

Due to susceptibility of the embryos to oxidative stress after zygote gene activation, increased oxidative stress leads to apoptosis of embryos (40). LC acts as an antioxidant and could protect cells and embryos from damage caused by oxidative stress, mitochondria dysfunction, and ultimately inhibition of apoptosis (41, 42). Apoptotic index was lower while the expression of the Bcl-xL was higher in blastocysts derived from BCB+ oocytes than those from BCB- ones (28). 

Thus, we assayed the expression of the Bcl-xL (an anti-apoptotic) and Bax (a pro-apoptotic) genes in blastocysts to determine whether apoptosis would be altered after treatment of BCB+ oocytes with LC. Our results showed that LC (0.3 or 0.6 mg/ml) added to IVM medium did not affect the level of Bax mRNA level in blastocysts. However, an increased expression of Bcl-xL was observed in blastocysts after treatment of oocytes with 0.6 mg/ml LC. It appears that there is a positive relationship between gene expression of Bcl-xL and the developmental competence in the embryos derived from oocytes treated with 0.6 mg/ml LC. The present findings are consistent with a previous report that supplementation of in vitro culture medium with LC (0.5 mg/ml) decreased the number of apoptotic cells in porcine activated blastocysts (41). It has been demonstrated that treatment of pig oocytes with LC (10 mM) increased the expression of both Bax and p-Bcl-xL mRNA in somatic cell nuclear transfer blastocysts. However, the cause of improved embryonic development despite increased expression of Bax mRNA after LC treatment was not clarified (30). Further investigations could elucidate the precise molecular mechanisms underlying LC function on developmental competence of oocytes and embryos.

Our study has some limitations that should be considered in the interpretation of the results. First, due to budget limitation, we did not measure genes expression at protein levels. Although alterations in mRNA levels do not necessarily reflect protein alterations, gene expression at mRNA level is still informative (43, 44). Second, in this study, we evaluated two apoptosis-related genes (i.e. Bax and Bcl-xL). Evaluation of other apoptosis-related genes, such as caspase-3, may increase the validity of our results. Studying the expression of other apoptosis-related genes should also be considered in our further study.

## Conclusion

Our results showed that treatment of BCB+ oocytes with LC during IVM ameliorates the reproductive success by regulating expression of genes involved in normal embryonic development. Because of the similarity of human preimplantation development with that in mouse, our data may illustrate a possible role for the LC in culture media of human BCB+ oocytes which needs to be further examined in future studies.
